# Development of a Cost-Effective and High-Fidelity Gel Model for Peripheral Ultrasound-Guided Intravenous Access Training

**DOI:** 10.7759/cureus.70181

**Published:** 2024-09-25

**Authors:** Colin Standifird, Hunter E Triplett, Charlton Bassett, Kaden Norman, Emily Ames, Joshua Levy, Marvi Moreno, Michael Lauria, Kathryn Sulkowski, Edward Simanton, Eugene Kang

**Affiliations:** 1 Emergency Medicine, Kirk Kerkorian School of Medicine at UNLV (University of Nevada, Las Vegas), Las Vegas, USA; 2 Emergency, Kirk Kerkorian School of Medicine at UNLV (University of Nevada, Las Vegas), Las Vegas, USA; 3 Radiology, University Medical Center of Southern Nevada, Las Vegas, USA; 4 Emergency Medicine, University of Washington, Seattle, USA; 5 Emergency Medicine, Mike O'Callaghan Military Federal Medical Center, Nellis Air Force Base, USA; 6 Medical Education, University of Nevada Las Vegas School of Medicine, Las Vegas, USA; 7 University of Nevada Las Vegas, Office of Medical Education, Las Vegas, USA; 8 Emergency Medicine, University Medical Center of Southern Nevada, Las Vegas, USA

**Keywords:** cost effective, difficult intravenous access, skills and simulation training, teaching technology, ultrasound-guided

## Abstract

Introduction

Simulation tools are crucial in medical education but current commercial models for ultrasound-guided intravenous (IV) access lack complexity and can be prohibitively expensive. This article proposes a cost-effective gel model system that replicates realistic vein and artery interactions, addressing the limitations of traditional models. An advanced gelatin model was constructed that incorporates intricate vein and artery configurations and enhances medical training by providing a more authentic experience. Patient testing will further validate its efficacy, promising improved accessibility for skill refinement in resource-constrained environments.

Methods

In a controlled study at the Kirk Kerkorian School of Medicine at the University of Nevada, Las Vegas, 12 medical students with limited ultrasound experience participated in a workshop using novel and Blue Phantom™ models for ultrasound-guided IV catheter placement. The advanced gelatin model, created with Ziplock™ Tupperware®, ultrasonography gel-filled balloons, and gelatin, proved more effective, as assessed by participants' post-training comfort levels. The comparison of participants’ pre- and post-training comfort levels with the models was the primary study objective. Participants were asked to complete a confidence survey based on a five-point Likert scale, and after using both models, this survey was re-administered to assess the participant's level of comfort after model use. The statistical analysis comparison of post-training survey data to the pre-training survey data was accomplished using SPSS version 29 (IBM Corp, Armonk, NY), where a paired t-test was set at a significance threshold of p <0.05.

Results

Analysis of data from both commercially made and advanced ultrasound-guided IV models using a paired t-test revealed a significant advantage for the advanced model. Participants, despite limited ultrasound experience, reported feeling over 4 points higher in skill confidence (p = 0.004) with the advanced model. Its popularity stems from diverse vasculature modeling, proving effective for both experienced practitioners and inexperienced individuals, maintaining value as user skill levels increase.

Conclusion

This study proposes an advanced model for ultrasound-guided peripheral IV access training, demonstrating a statistically significant increase in confidence levels. Despite limitations such as small sample size and single-site participation, the advanced model's adaptability and cost-effectiveness make it a strong contender for replacing current commercial models, potentially enhancing proficiency and confidence while reducing costs.

## Introduction

Specialists across many fields attribute their expertise to years of deliberate practice. However, exposures to procedures are often limited by rare pathology and patient presentations. Throughout medical education, simulation tools have been an integral part of procedural training. Artificially creating scenarios allows providers the opportunity to experience phenomena that could exist in reality [1]. Simulation offers a supportive, reproducible, and standardized environment [2]. Ethical considerations, increasingly stringent medical education requirements, and technological advancements in medicine have made simulation a priority in medical training [3].

Skill proficiencies required for ultrasound (US) training and education are outlined by the American Society of Regional Anaesthesia and Pain Medicine and the European Society of Regional Anaesthesia and Pain Therapy [4]. They include understanding the operation of the US machine, image acquisition, optimization and interpretation, needle insertion, and needle injection. The American College of Emergency Physicians also notes the importance of site selection, positioning, and technique used to visualize needle insertion and confirm needle placement for ultrasound-guided intravenous (USIV) access [4,5]. While these skills can be practiced on patients or live tissue models, simulation allows for a safe, economical, and efficient alternative [5,6]. There is growing evidence that supports the valuable role of simulation in procedural skill acquisition, such as the performance of USIV access [7,8].

Traditionally, gel models have been used for USIV simulation and training. However, available commercial models impose limits due to their high cost and simplicity [5,9,10]. These commercial models lack the ability to accurately resemble the complex vasculature found in real-world practice. This article addresses this problem by introducing a novel method of creating gel models that economically incorporates customizable and configurable vessels that closely resemble the variability seen anatomically. Gottlieb et al. suggest that veins measuring more than 0.4 cm in diameter are associated with higher rates of cannulation in patients. Most commercial models include vessel sizes greater than 1 cm on average [4]. We aim to introduce a novel technique to practice USIV cannulation with gel models. Our purpose is to assess the differences in provider procedural confidence between the traditional commercial vs the advanced gelatin model for placing US-guided peripheral IVs [9]. 

This article was previously represented as a meeting poster at the 2023 Nevada State Medical Association Annual Conference on August 26th, 2023, at the 2024 Southwest Regional Emergency Medicine Conference on January 26th, 2024, at the 2024 American Institute of Ultrasound in Medicine Ultracon Conference on April 7th, 2024, at the 2024 Western Group Educational Affairs Regional Conference on May 5th, 2024, and at the 2024 International Association of Medical Student Educators Conference on June 16th, 2024.

## Materials and methods

**Figure 1:**
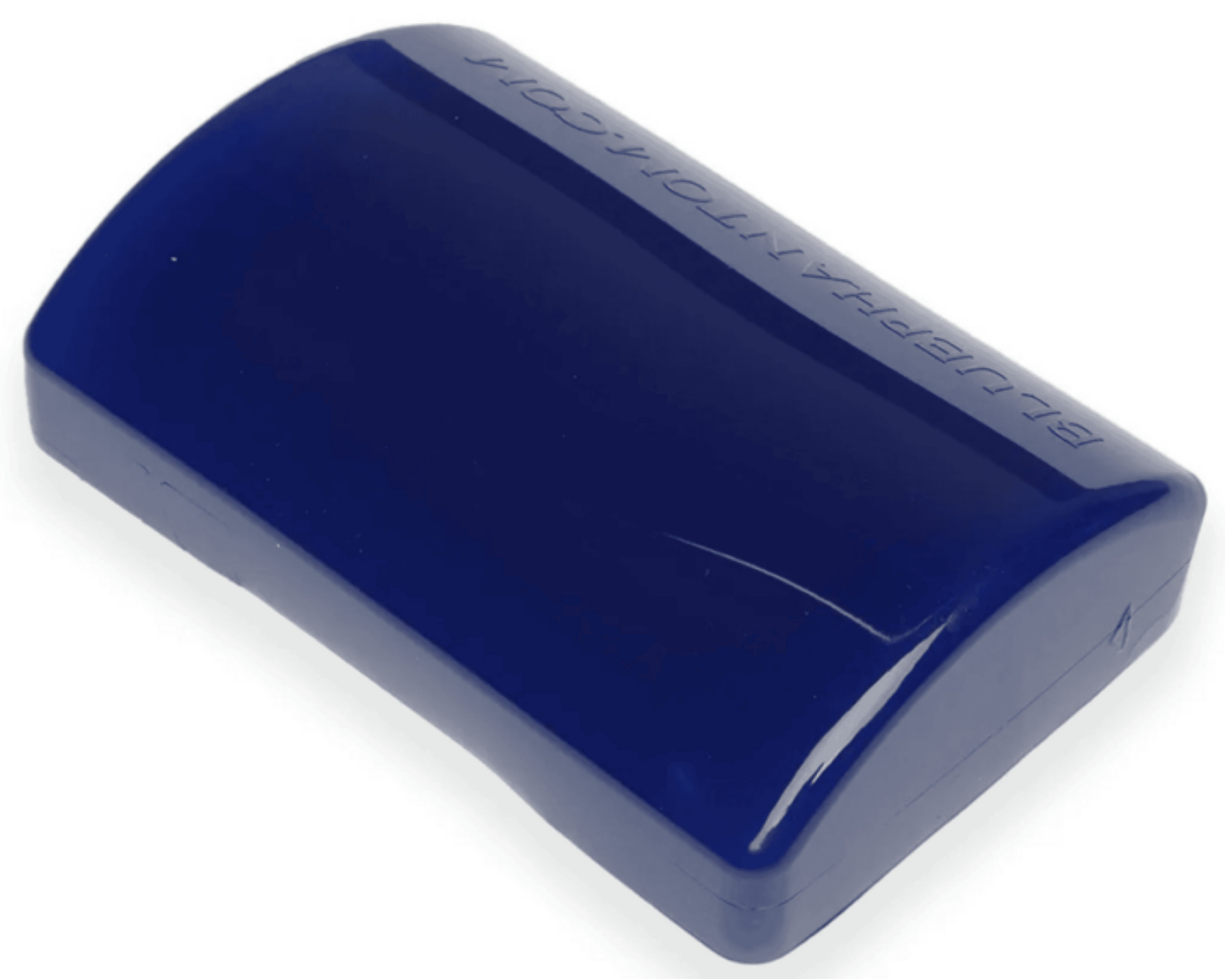
Depicts the commercially available Blue Phantom™ model used in the study. The website for the Blue Phantom™ model can be found in Ref. [11].

This study employed two models. The experimental model, referred to as the advanced gelatin model, and the control model, referred to as the commercially available Blue Phantom™ model [11](Figure 1).

**Figure 2:**
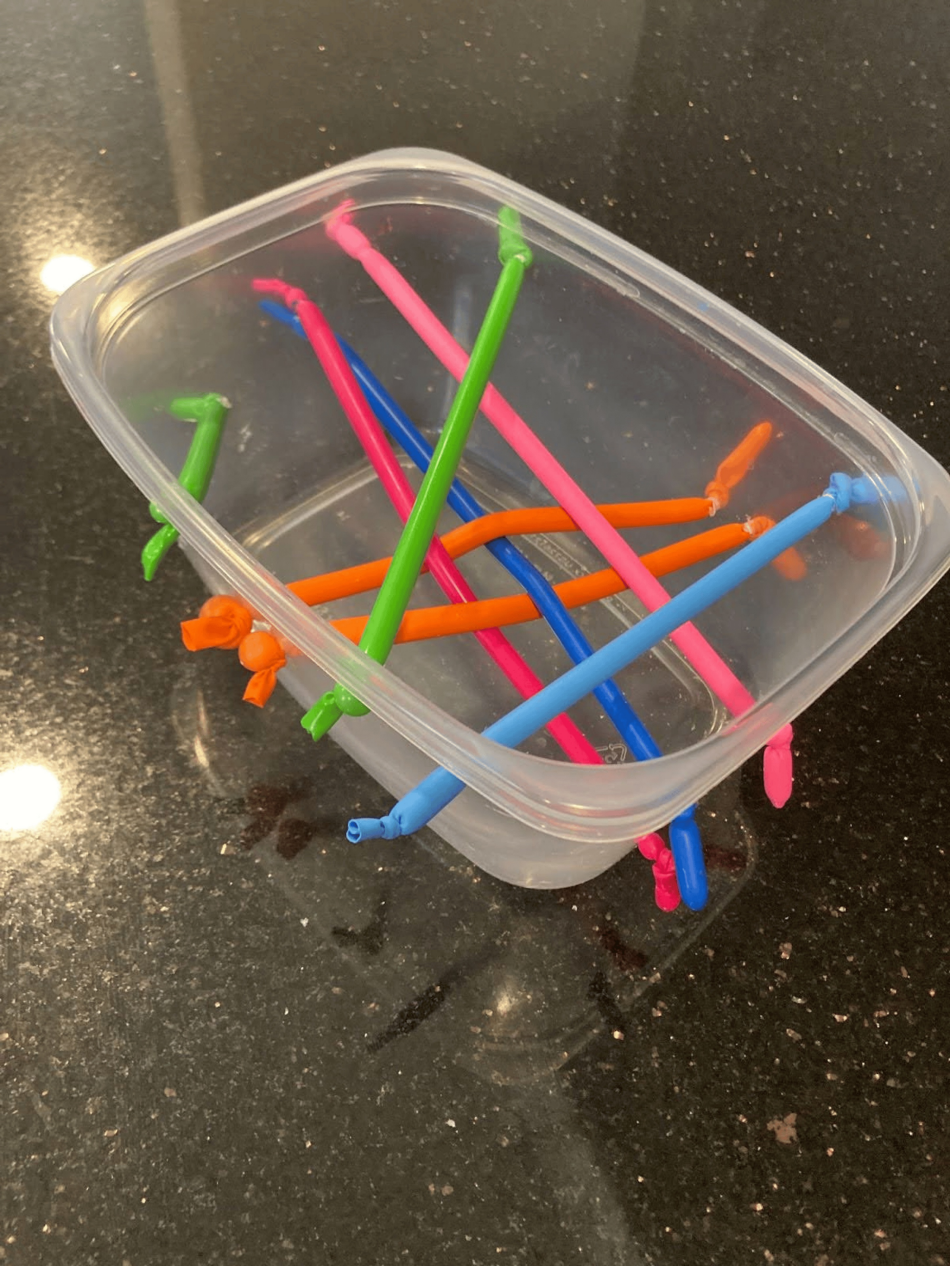
Depicts the advanced gelatin model used in the study before pouring gelatin. This figure shows the advanced gelatin model used in the study before the gelatin was poured. This figure demonstrates the structure of the model, including how the vasculature was modeled with balloons and how the model design incorporates complex, interwoven vessel configurations.

**Figure 3:**
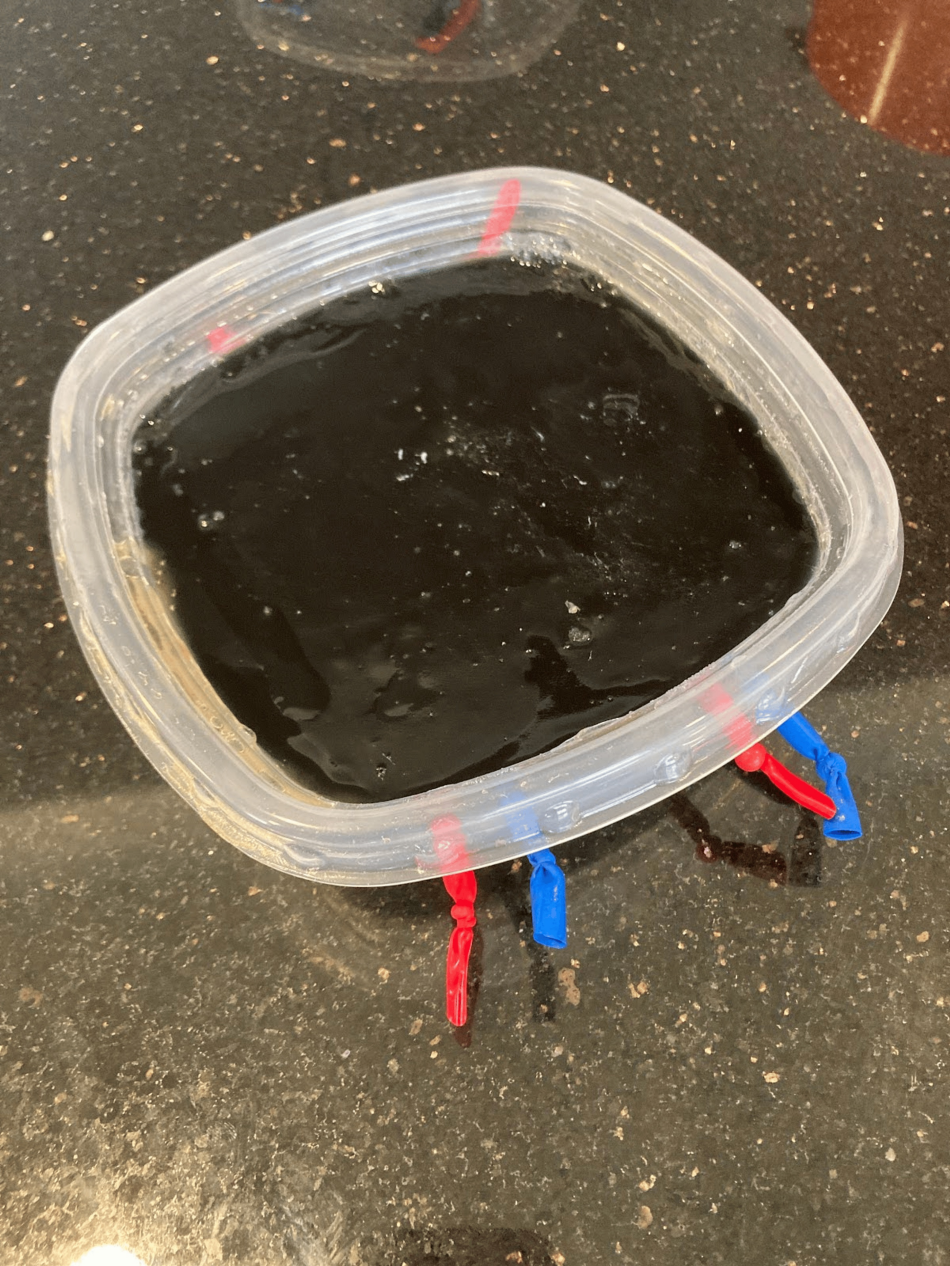
Depicts the advanced gelatin model after the gelatin has been poured. This figure depicts the advanced gelatin model after the gelatin has been poured and hardened. This figure demonstrates the opacity of the model and its complete construction for use in the study.

For the advanced gelatin model design, a good-grade disposable plastic storage container (Tupperware®) was used as the frame to hold the gelatin mixture and the simulated vessels (Figure 2). To simulate peripheral vessels, thin commercially available latex balloons were filled with colored ultrasonography gel, matched with the balloon color, and tied off at the open end. The average vessel size was 0.5 cm and placed at depths ranging from 0.3 to 1.5 cm, which are depths associated with higher cannulation rates. Five vessels were arranged side by side, stacked, and crossed in vein-over-artery and artery-over-vein configurations. Holes were drilled in the side of a good-grade disposable plastic storage container (Tupperware®) and the gel-filled balloons were inserted in the conformations detailed above (Figure 2). The food-grade gelatin used in the gelatin models was combined with cold water first, then with boiling water and a commercially available psyllium fiber supplement (Metamucil™). After stirring the liquids thoroughly, allowing all the particles to dissolve, the mixture was strained twice and then poured into the good-grade disposable plastic storage container (Tupperware®). The gelatin was then allowed to harden at 40 degrees Fahrenheit for 24 hours. The final product was opaque (Figure 3). For this research project, a total of two models were used - one advanced gelatin model and one commercial Blue Phantom™ model [11]. The commercially produced Blue Phantom™ model was provided by KKSOM [11]. 

Participants practiced inserting catheters into fictitious peripheral veins with each of the two models using 18 gauge, 2.5 inch IV catheters and ultrasonography gel. Before using either of the models, participants filled out a pre-survey assessing their comfort level placing USIV catheters using a five-point Likert scale. Participants utilized both portable handheld Butterfly iQ+™ probes as well as standard Mindray™ linear probes [12,13]. The participants' subjective assessment of which models they felt more confident using pre- and post-training served as the study's primary objective. Participants were trained using a standardized US peripheral IV insertion curriculum that encouraged the use of short-axis and long-axis techniques to insert an IV catheter under US guidance. Participants used both the advanced gelatin model and the commercial Blue Phantom™ model presented in random order [11]. There was no time limit for participants to practice. After participants felt they had sufficient training, they were asked to fill out a survey using a QR code located beside the model. After using both of the models, the original survey was re-administered as a post-survey in order to re-assess the participant’s level of comfort using the same five-point Likert scale. The survey is included in the appendix for review. The statistical analysis comparison of post-training survey data to the pre-training survey data was accomplished using SPSS version 29 (IBM Corp, Armonk, NY), where a paired t-test was set at a significance threshold of p <0.05. This study was reviewed and deemed exempt by the Institutional Review Board of the University of Nevada, Las Vegas (IRB Exemption Number UNLV-2023-249).

This was a controlled study conducted at the Kirk Kerkorian School of Medicine at the University of Nevada, Las Vegas (KKSOM) during a physician-led US workshop. Our sample participants included six first-year medical students, five second-year medical students, and one third-year medical student, for a total participant sample of N = 12. This study examined a convenience sample of medical students attending the KKSOM participating in the US skills workshop.

## Results

**Figure 4:**
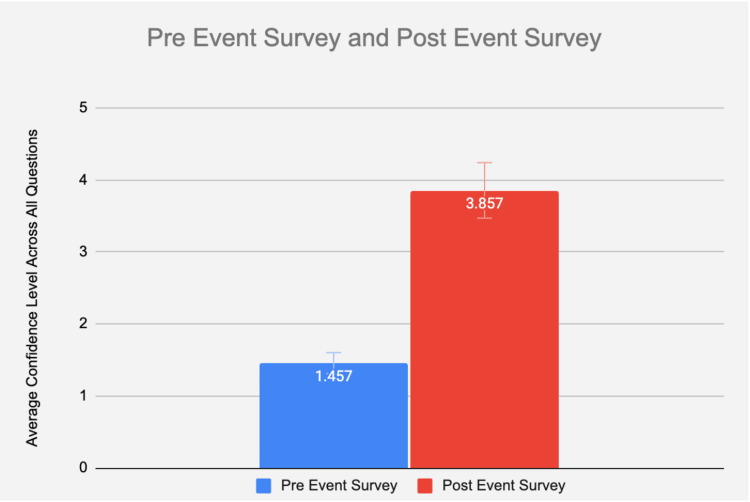
Depicts the average improvement in confidence levels for event participants, irrespective of model type. The data have been represented as mean ± SD.

**Figure 5:**
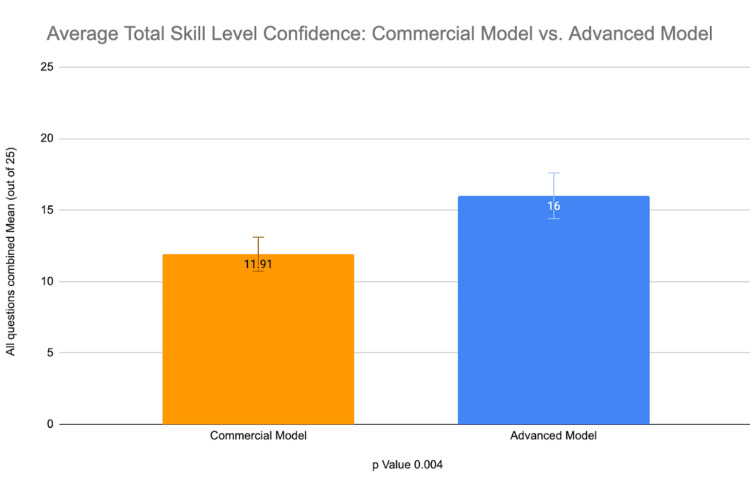
Depicts the average total skill level confidence defined as the sum of responses for the surveys of each model. The data have been represented as mean ± SD.

**Figure 6:**
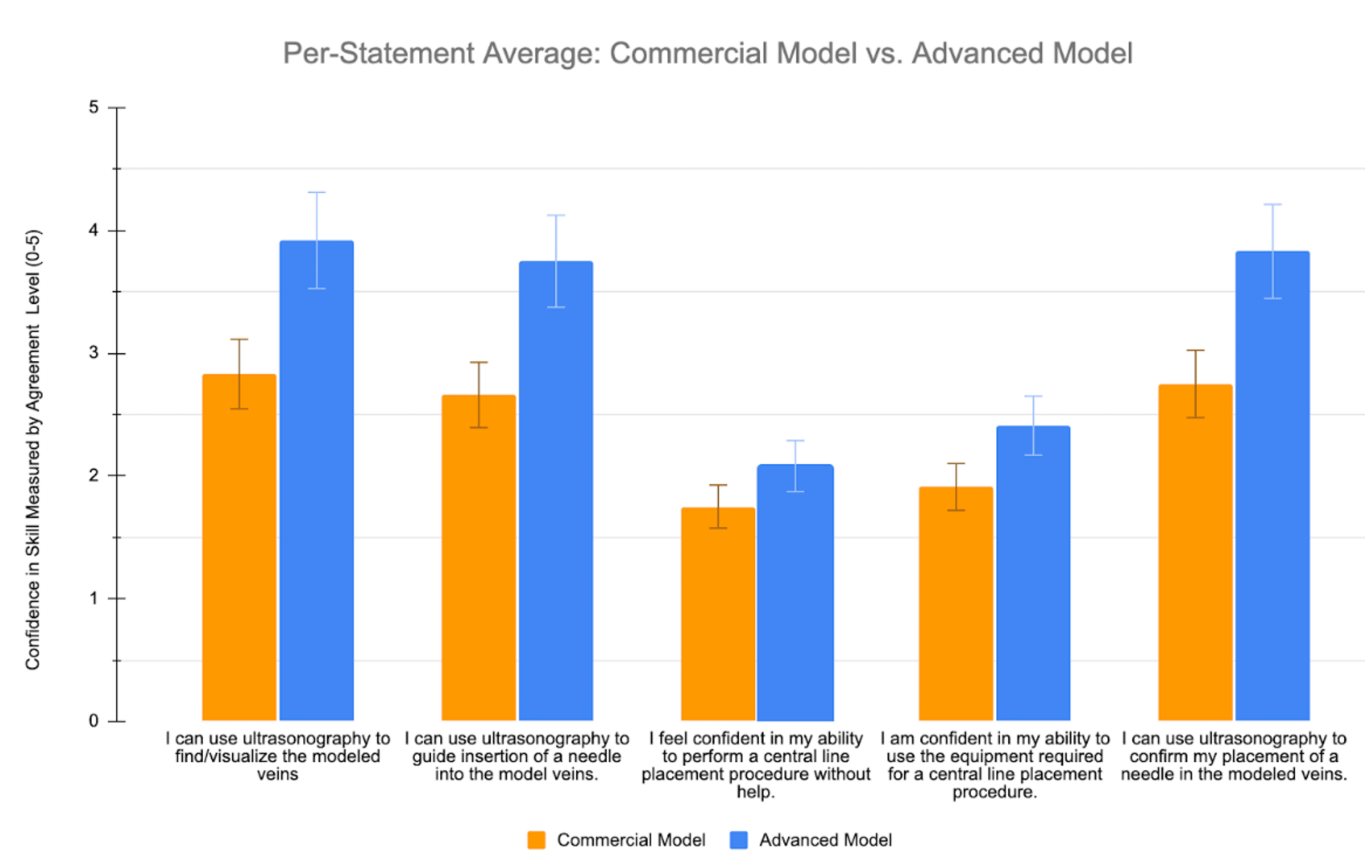
Depicts the average skill confidence levels for each model organized by statement on the survey. The data have been represented as mean ± SD.

Survey confidence data from the commercially made USIV model and the advanced gelatin USIV model were compiled into spreadsheets and assessed through analytical statistics. A paired t-test was used to compare the values of the two models. This analysis was used to determine statistical significance in the perceived confidence in USIV skills for the study participants. Before the event, the participants reported an average mean confidence in skill level (measured through the degree of agreement to each of the survey questions) of 1.457. The mean confidence level of the participants rose by 2.4 (Figure 4) to about 3.9. Data suggest that the advanced gelatin model made users feel, on average, more than 4 total points higher in skill confidence levels for the advanced gelatin model when compared to the commercial model (p = 0.004). Our data demonstrated a statistically significant advantage for participants when using the advanced USIV model over the commercial model (Figure 5). While the advanced gelatin model did produce higher average skill confidence levels for each statement, some statements had more significant differences between the commercial model and the advanced gelatin model than others. Statement 1 (I can use ultrasonography to find/visualize the modeled veins), statement 2 (I can use ultrasonography to guide insertion of a needle into the model veins), and statement 5 (I can use ultrasonography to confirm my placement of a needle in the modeled veins) all had statistically significant differences (p = 0.003, 0.01, and 0.008, respectively) between their respective commercial and advanced gelatin model surveys. Statement 4 (I am confident in my ability to use the equipment required for a central line placement procedure) had an insignificant difference of 0.5 points between the two (p = 0.54), followed by statement 3 (I feel confident in my ability to perform a central line placement procedure without help) with the smallest insignificant difference of 0.3 (p = 0.68) between the two (Figure 6). The original, complete survey has been included in the appendix. 

## Discussion

In general, participants’ confidence values rose by an average of 250% after the training workshop. More specifically, the advanced gelatin model was preferred by the study participants over the Blue Phantom™ commercial model in all surveyed categories, but it was particularly favored for using ultrasonography to find/visualize the modeled veins, to guide the insertion of a needle into the model veins, and to confirm the placement of a needle in the modeled veins. The positive response toward the advanced gelatin model is most likely due to the more diverse modeled vasculature, with vessels demonstrating different diameters, being found at different depths, and having challenging vascular arrangement to work with. Although it would seem intuitive that a complicated model of this design would only be useful for training experienced practitioners, the surveyed average total skill confidence was 4 points higher in the advanced gelatin model than the Blue Phantom™ commercial model, with responses coming from a participant pool that was composed exclusively of medical students with very little experience. This suggests that the advanced gelatin model is a more effective tool for training inexperienced individuals than the Blue Phantom™ commercial model. Additionally, the modularity of the advanced model allows for increasing complexity to match the progression of the user's skill level. Importantly, the advanced gelatin model costs approximately $5.08 to make, which is less than 1% of the average cost of the Blue Phantom™ commercial models, which cost approximately $700 each (prices in US dollars as of September 2024) [9]. These characteristics, with specific emphasis on the substantially lower cost, all make the advanced gelatin model a viable alternative for US-guided peripheral IV access training. Future research studies are needed to evaluate the effectiveness of cannulation among more skilled practitioners using each model to further determine the benefits of each model. Also, further research needs to be done to determine if confidence values are associated with better cannulation rates/outcomes in clinical scenarios.

The results of this study are limited by the small number of participants recruited from a single institution and who were surveyed at a single event. This limited sample size could reduce the generalizability of our study, and potentially reduce the statistical power of our results. Expanding the participant pool to other institutions and expanding to include residents, fellows, and attending physicians would allow for a more stratified analysis of the participant pool statistics, reduce subliminal biases, and increase the generalizability of the study. Another limitation of this study is that it did not examine the long-term viability of the advanced gelatin model compared to the commercial model, in terms of how many needle sticks can be performed before the models break down. This type of structural integrity is important when comparing training tools and will be important for further research. A final limitation of this study is that it was performed using a limited five-question survey based on the subjective evaluation of the participants. This type of measurement allows for response bias on behalf of the participants, particularly because the study did not include an expert’s objective assessment of participant competency when using the different models. Although the qualitative questionnaire utilized was pilot-tested by the authors prior to the study, it was not validated.

## Conclusions

This controlled study conducted by the medical students at the Kirk Kerkorian School of Medicine at the University of Nevada, Las Vegas proposes an alternative, advanced gelatin model to the Blue Phantom™ commercial models currently used for USIV peripheral access training. Study results found a statistically significant increase in skill confidence levels for each survey question, highlighting the effectiveness of using the more complex, advanced gelatin model for USIV training. ​When examining the survey results further, study participants expressed notably higher confidence levels in their ability to use the US for vein visualization, needle insertion guidance, and needle placement confirmation when using the advanced gelatin model. These results counter assumptions that may exist about the need to train students who have little experience with simple models that lack complexity. The advanced gelatin model’s adaptability to users with multiple skill levels coupled with its cost-effectiveness compared to the current Blue Phantom™ commercial model make it a strong contender to replace the use of the currently available commercial model for USIV training.

## Appendices

**Table 1:** Complete survey provided to study participants. The data collected from the responses to this survey were used for all statistical analyses which served as the primary endpoint of the study.

Please answer the following questions with 0 being STRONGLY DISAGREE and 5 being STRONGLY AGREE: I can use ultrasonography to find/visualize the modeled veins	0	1	2	3	4	5
Please answer the following questions with 0 being STRONGLY DISAGREE and 5 being STRONGLY AGREE: I can use ultrasonography to guide insertion of a needle into the model veins.	0	1	2	3	4	5
Please answer the following questions with 0 being STRONGLY DISAGREE and 5 being STRONGLY AGREE: I feel confident in my ability to perform a central line placement procedure without help.	0	1	2	3	4	5
Please answer the following questions with 0 being STRONGLY DISAGREE and 5 being STRONGLY AGREE: I am confident in my ability to use the equipment required for a central line placement procedure.	0	1	2	3	4	5
Please answer the following questions with 0 being STRONGLY DISAGREE and 5 being STRONGLY AGREE: I can use ultrasonography to confirm my placement of a needle in the modeled veins.	0	1	2	3	4	5
